# RESILIENCE IN JAPANESE OLDER IMMIGRANTS AND ROLES OF COMMUNITY SUPPORT DURING THE COVID-19 PANDEMIC

**DOI:** 10.1093/geroni/igad104.2901

**Published:** 2023-12-21

**Authors:** Mineko Wada, Sarah Canham

**Affiliations:** Hiroshima University, Hiroshima, Hiroshima, Japan; University of Utah, Salt Lake City, Utah, United States

## Abstract

The objective of this community-based study was to explore how Japanese older immigrants cultivated resilience in overcoming challenges during the COVID-19 pandemic and how Tonari Gumi, a community service agency, supported the process. As Japanese people make up a small proportion of the population in Canada, there are limited resources to meet their distinct needs. Thus, Japanese older adults were particularly affected by disrupted support and service systems when COVID-19 public health orders were implemented. In this qualitative study, seven community-dwelling Japanese older immigrants and five staff from Tonari Gumi participated in semi-structured interviews. The interviews were analyzed thematically using a conceptual lens of resilience, which refers to the ability to survive and thrive in the face of adverse life experiences. Our analysis yielded three themes: Challenges and concerns; Staying active: physically, mentally, and socially; and Creating needs-based services and programs to survive, connect, and enjoy. The initial challenge experienced by Japanese older immigrants was “a feeling of emptiness,” followed by hardships associated with digital literacy, English literacy, fear of COVID, and concerns about the future. In response to the challenges, Japanese older immigrants stayed active by rebuilding and sustaining regular exercise habits, nurturing and sustaining positive mindsets, and sustaining and expanding social connections. Tonari Gumi developed and delivered a variety of new services and programs to meet the needs of survival, social connection, and fun. We will discuss key actions that older individuals and service providers took on to facilitate resilience and implications for research, policy, and practice.

